# Optimizing antimicrobial prescribing: Are clinicians following national trends in methicillin-resistant *staphylococcus aureus* (MRSA) infections rather than local data when treating MRSA wound infections

**DOI:** 10.1186/2047-2994-2-28

**Published:** 2013-10-15

**Authors:** Marin L Schweizer, Eli N Perencevich, Michael R Eber, Xueya Cai, Michelle D Shardell, Nikolay Braykov, Ramanan Laxminarayan

**Affiliations:** 1 Iowa City VA Health Care System, Iowa City, IA, USA; 2 Division of General Internal Medicine, Carver College of Medicine, University of Iowa, Iowa City, IA, USA; 3 Center for Disease Dynamics, Economics & Policy, Washington, DC, USA; 4 Department of Biostatistics and Computational Biology, University of Rochester, Rochester, NY, USA; 5 Department of Epidemiology and Public Health, University of Maryland School of Medicine, Baltimore, MD, USA; 6 Princeton University, Princeton, NJ, USA; 7 Public Health Foundation of India, New Delhi, India

**Keywords:** Drug utilization, Antimicrobial prescribing, Methicillin-resistant *Staphylococcus aureus*

## Abstract

**Background:**

Clinicians often prescribe antimicrobials for outpatient wound infections before culture results are known. Local or national MRSA rates may be considered when prescribing antimicrobials. If clinicians prescribe in response to national rather than local MRSA trends, prescribing may be improved by making local data accessible. We aimed to assess the correlation between outpatient trends in antimicrobial prescribing and the prevalence of MRSA wound infections across local and national levels.

**Methods:**

Monthly MRSA positive wound culture counts were obtained from The Surveillance Network, a database of antimicrobial susceptibilities from clinical laboratories across 278 zip codes from 1999–2007. Monthly outpatient retail sales of linezolid, clindamycin, trimethoprim-sulfamethoxazole and cephalexin from 1999–2007 were obtained from the IMS Health Xponent^TM^ database. Rates were created using census populations. The proportion of variance in prescribing that could be explained by MRSA rates was assessed by the coefficient of determination (R^2^), using population weighted linear regression.

**Results:**

107,215 MRSA positive wound cultures and 106,641,604 antimicrobial prescriptions were assessed. The R^2^ was low when zip code-level antimicrobial prescription rates were compared to MRSA rates at all levels. State-level prescriptions of clindamycin and linezolid were not correlated with state MRSA rates. The variance in state-level prescribing of clindamycin and linezolid was correlated with national MRSA rates (clindamycin R^2^ = 0.17, linezolid R^2^ = 0.22).

**Conclusions:**

Clinicians may rely on national, not local MRSA data when prescribing clindamycin and linezolid for wound infections. Providing local resistance data to prescribing clinicians may improve antimicrobial prescribing and would be a possible target for future interventions.

## Background

Methicillin-resistant *Staphylococcus aureus* (MRSA) is a major cause of outpatient wound infections, especially in the era of community-associated MRSA [1-10]. In the outpatient setting, physicians often prescribe antimicrobials for wound infections before culture results are known. The Infectious Diseases Society of America (IDSA) and U.S. Centers for Disease Control and Prevention recommend empirical coverage aimed at community-associated MRSA for outpatient wound infections if MRSA is common in that community. This empiric coverage includes clindamycin, trimethroprim-sulfamethoxazole, or linezolid [2-7]. The CDC specifically recommends that healthcare facilities know infection and resistance trends in their facility and facilities nearby to fight the spread of resistance [5,6].

When prescribing empiric antibiotic therapy, clinicians may be influenced by many factors including patient symptoms, patient risk factors, and the prevalence of MRSA nationally or in their community. This study aims to assess whether variation in prescribing practice is influenced by local, state or national prevalence of MRSA. Antibiotic-resistance rates at the national level may not correlate with local resistance rates under which antimicrobials are prescribed empirically. Local rates of MRSA infections could help determine the probability that the current patient is infected with MRSA. Yet, if these rates are not known or difficult to access, clinicians may be forced to rely solely on national MRSA trends.

Prior studies have found that local and national prescribing of anti-MRSA antimicrobials increased after the introduction of community-associated MRSA wound infections [7-10]. However, those studies also found that many patients received empiric therapy with antibiotics that were inactive against locally circulating strains of MRSA [7,8].

The present study compared outpatient trends in antimicrobial prescribing and the extent of their correlation with the prevalence of MRSA wound infections across local (i.e. zip code, state), and national levels. If local prescribing does not appear to be correlated with local rates of MRSA infections, then this may identify an opportunity for future intervention trials that assess the benefit of providing clinicians with local antibiogram data.

## Materials and Methods

### Sources of data

Data from two different databases were compared. Data on monthly outpatient prescriptions per zip code were obtained for the period of January 1999 to December 2007 from the IMS Health Xponent^TM^ database. The IMS Health Xponent^TM^ database tracks more than 70% of all outpatient prescriptions in the United States using transaction records at retail pharmacies, and uses a patented projection methodology to represent 100% coverage of all prescription activity. Monthly outpatient prescription rates were calculated for clindamycin, linezolid, and trimethoprim-sulfamethoxazole. We chose these antimicrobials because they are recommended for outpatient empiric therapy for suspected MRSA wound infections [2,3]. Cephalexin, an antimicrobial to treat methicillin-susceptible *S. aureus* infections, was assessed to determine whether rates of cephalexin prescribing decreased when rates of MRSA infections increased.

Data on monthly outpatient MRSA wound infections per laboratory zip code for the period of January 1999 to December 2007 were collected from The Surveillance Network (TSN; Eurofins Medinet, Herndon, VA). TSN is a nationally and regionally representative database of bacterial species identification and antimicrobial susceptibility results gathered from 300 US hospitals among 278 zip codes [11,12]. Participating laboratories are geographically dispersed and constitute a nationally representative sample based on hospital bed size and patient population. These laboratories are required to submit all bacterial isolates to TSN. Only laboratories that are certified by the Clinical Laboratory Standards Institute (CLSI) and that report on the basis of CLSI reference methods are included in TSN [13]. Results were filtered to remove repeat isolates.

MRSA infection was defined as a *S. aureus* positive culture resistant to oxacillin. Only outpatient MRSA wound infections from the TSN database were included in this study. These included surgical wounds, soft tissue infections and infections associated with intravascular devices and catheters. Data from the US Census 2000 were used to assess the population of each zip code included in each study.

### Statistical analysis

Monthly MRSA-positive wound culture rates were compared to rates of antimicrobial prescribing at the zip code, state and national levels. Zip code level rates were calculated using the US census 2000 populations for each zip code. The denominator for each state level rate was the sum of the zip code populations included in this study for each state. The denominator for the national level rates was the sum of the US census 2000 populations for each of the 278 zip codes included in this analysis. The proportion of variance in prescribing that could be explained by MRSA rates was assessed by the coefficient of determination (R^2^), using population weighted linear regression [14].

The coefficient of partial determination was calculated to measure the marginal effect of MRSA infection when time was already accounted for in the model [14]. To calculate the coefficient of partial determination, a full model of the log-transformed rate of antimicrobial prescriptions was first estimated when time, MRSA infection, and the interaction of time with MRSA infection were considered explanatory variables. A reduced model of log-transformed rate of antimicrobial prescriptions with time only was then estimated. The relative marginal reduction in the variation of antimicrobial prescriptions between the two models was calculated to measure the additional contribution of MRSA infection to the antimicrobial prescriptions. All analyses were performed using SAS software (SAS Institute, Cary, NC) version 9.2.

### Ethics

The funding source, Robert Wood Johnson Foundation, had no role in study design; collection, analysis, and interpretation of data; in the writing of the report; or the decision to submit the paper for publication. This study deemed to be not human subjects research and IRB approval was waived by the University of Iowa IRB. Patient consent was also waived by the University of Iowa IRB as the data were deidentified and previously collected.

## Results

From January 1, 1999 to December 31, 2007 there were 13,295 prescriptions for clindamycin, 10,973 prescriptions for linezolid, 8,307 prescriptions for trimethoprim-sulfamethoxazole and 3,022 prescriptions for cephalexin in the IMS Health Xponent^TM^ database. The national rates of clindamycin and linezolid prescribing increased over time (p < 0.01). The national rates of cephalexin prescribing decreased over time (p < 0.01).

There were 107,215 MRSA positive wound cultures in TSN database. The national rates of MRSA wound infections increased from 23.9 MRSA infections per million people in January 1, 1999 to 118.7 MRSA infections per million people in December 31, 2007 (p < 0.01). Antimicrobial prescribing at the zip code level was not correlated with zip code, state or national MRSA infection rates (R^2^ < 0.10) for clindamycin, linezolid, trimethoprim-sulfamethoxazole or cephalexin. The correlations between zip code level prescribing and zip code level infection rates are presented in Table 1.

**Table 1:** **Proportion of variance (r**^**2**^**) in zip code level antimicrobial prescribing that could be explained by zip code level MRSA wound infection rates**

**Zip code level antimicrobial prescribing rate**	**Zip code level infection rate (R**^**2**^**)**^**a**^	**Zip code level infection rate**
		**(Coefficient of partial determination)**^**b**^
**Trimethoprim-sulfamethoxazole**	0.04	0.03
**Cephalexin**	0.05	0.03
**Linezolid**	0.09	0.09
**Clindamycin**	0.06	0.03

^a^R^2^ represents the unadjusted proportion of variance in prescribing that could be explained by MRSA rates using population weighted linear regression; ^b^Coefficient of Partial Determination represents the marginal effect of MRSA infection on prescribing when time was already accounted for in the model.

Source: Author’s calculations with susceptibility information from The Surveillance Network® (TSN) and prescription data derived from IMS Health Xponent™ January 1999-December 2007, IMS Health Incorporated. All Rights Reserved.

**Figure 1:**
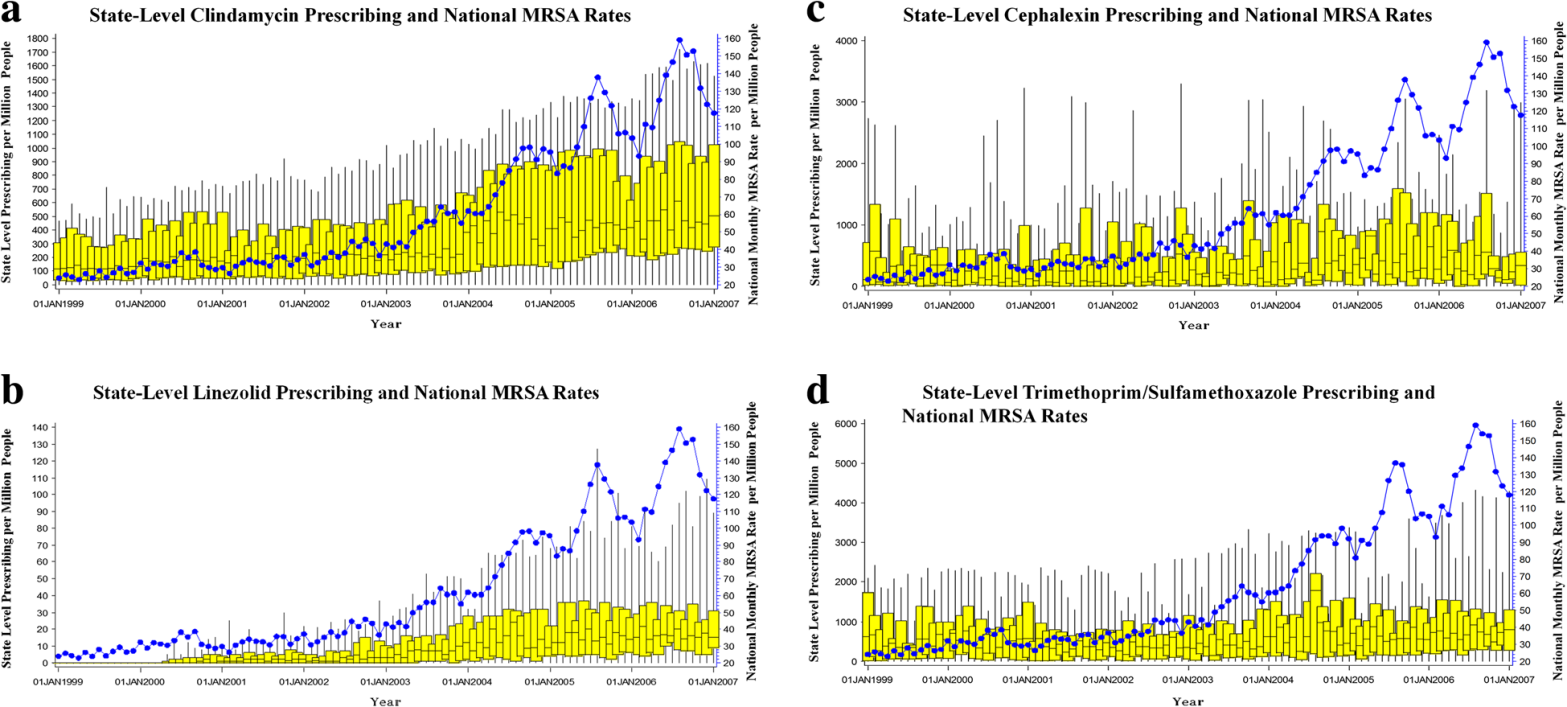
**State Level Prescribing and National MRSA Rates. a**. State-level clindamycin prescribing vs. national MRSA rates. **b**. State-level linezolid prescribing vs. national MRSA rates. **c**. State-level cephalexin prescribing vs. national MRSA rates. **d**. State-level trimethoprim/sulfamethoxazole prescribing vs. national MRSA rates. Note: Box and whisker plots represent median, interquartile range and whisker boundaries of monthly antimicrobial prescribing among all states in the IMS Health Xponent^TM^ database; line represents monthly national MRSA rates. Source: Author’s calculations with susceptibility information from The Surveillance Network® (TSN) and prescription data derived from IMS Health Xponent™ January 1999-December 2007, IMS Health Incorporated. All Rights Reserved.

State level prescriptions of trimethoprim-sulfamethoxazole and cephalexin were not well described by state or national rates of MRSA (R^2^ < 0.01). State level prescriptions of clindamycin were not correlated with state level MRSA rates (R^2^ < 0.01). State level prescriptions of linezolid were also not correlated with state MRSA rates (R^2^ = 0.004). The variance in state level prescribing of clindamycin and linezolid was best explained by national MRSA rates (clindamycin R^2^ = 0.17; linezolid R^2^ = 0.22) (Table 2). Thus, the proportion of variance in state-level prescribing of clindamycin that can be described by national MRSA wound infection rates is 17% and the proportion of variance of linezolid prescribing that can be described by national MRSA wound infection rates is 22%. State level prescribing for trimethoprim-sulfamethoxazole, cephalexin, clindamycin and linezolid and national MRSA wound infection rates are graphically represented in Figure 1.

**Table 2:** **Proportion of variance (R**^**2**^**) in state level antimicrobial prescribing that could be explained by MRSA wound infection rates at the state and national levels**

**State level**	**State level**	**National level**	**State level infection**	**National level infection**
**antimicrobial**	**infection rate (R**^**2**^**)**^**a**^	**infection**	**rate (Coefficient of**	**rate (Coefficient of**
**prescribing rate**		**rate (R**^**2**^**)**^**a**^	**partial determination)**^**b**^	**partial determination)**^**b**^
**Trimethoprim-sulfamethoxazole**	<0.001	0.03	0.10	0.004
**Cephalexin**	<0.001	0.01	0.08	0.003
**Linezolid**	0.004	0.22	0.004	0.22
**Clindamycin**	<0.001	0.17	0.10	0.008

^a^R^2^ represents the unadjusted proportion of variance in prescribing that could be explained by MRSA rates using population weighted linear regression; ^b^Coefficient of Partial Determination represents the marginal effect of MRSA infection on prescribing when time was already accounted for in the model.

Source: Author’s calculations with susceptibility information from The Surveillance Network® (TSN) and prescription data derived from IMS Health Xponent™ January 1999-December 2007, IMS Health Incorporated. All Rights Reserved.

The proportion of variance in cephalexin and trimethoprim-sulfamethoxazole prescribing explained by infection rates remained low in the coefficient of partial determination models. For rates of linezolid prescribing, the coefficient of partial determination model that accounted for the interaction between time and infection rates resulted in the same proportion of variance in prescribing explained by infection rates as the unadjusted model (state level infection rate R^2^ = 0.004, national level infection rate R^2^ = 0.22). However, the proportion of variance in clindamycin prescribing described by national MRSA infection rates decreased substantially (R^2^ = 0.007) (Table 2).

## Discussion

Over the nine-year study period, we found that state-level clindamycin and linezolid rates are correlated with national MRSA wound infection rates, but not correlated with zip code or state level MRSA wound infection rates. These results suggest that national, not local, MRSA data may be influencing clinicians’ decisions when prescribing empiric antimicrobials in the outpatient setting. This is at odds with CDC recommendations that facilities keep track of local rates of resistant infections in order to fight the spread of MRSA [4-6]. Thus, efforts by hospitals or health departments to share state or local MRSA rates with clinicians may represent an opportunity to improve antimicrobial prescribing and antimicrobial stewardship in general.

Local knowledge of MRSA infection and resistance trends can improve outpatient antibiotic prescribing. For example, a study by Marra et al., found that outpatient clinicians increased their use of clindamycin and trimethoprim-sulfamethoxazole after a regional (Western Canada) epidemic of community-associated MRSA. Yet the clinicians in that region also frequently prescribed clindamycin even though the predominant MRSA strain in that region was resistant to clindamycin [7]. Similarly, Gupta et al. found that although local rates of community-associated MRSA skin and soft tissue infections were increasing, the majority of patients with these infections were still receiving inactive antimicrobial therapy [8]. A good example of reporting MRSA rates at a regional level is the European Antimicrobial Resistance Surveillance System (EARSS) in which individual countries share their MRSA rates [15]. If other regions such as U.S. states or Canadian provinces similarly shared their MRSA rates, local clinicians would be able to use those rates to enhance antimicrobial prescribing.

This study found no correlation between zip code level antimicrobial prescribing and rates of MRSA wound infections. This could be due to two factors. First, clinicians may not have knowledge of zip code level rates of MRSA wound infections. However, it is likely that the zip code was too small of a unit of analysis. Zip code level rates of antimicrobial prescribing and infection data varied greatly from month to month. For instance, in one zip code the MRSA infection rate varied from 6.0 MRSA infections per 10,000 people in one month to 2.7 per 10,000 people in the following month. Fluctuations such as this would be difficult to correlate with any other measure. The zip code data are also limited because we assessed pharmacy zip codes and laboratory zip codes but we did not have access to the patients’ zip codes. It is possible that these zip codes may not coincide with a patient’s own zip code.

Rates of trimethoprim-sulfamethoxazole prescribing were not associated with MRSA wound infection rates at the zip code, state and national level. This is most likely because trimethoprim-sulfamethoxazole is prescribed for a variety of pathogens in the outpatient setting and is not as well correlated with MRSA infections as linezolid or clindamycin, which are prescribed for a fewer number of indications [16]. It is interesting to note that when time was adjusted for in the statistical models, the correlation between national MRSA infection rates and linezolid prescribing remained, but the correlation between national MRSA infection rates and clindamycin prescribing decreased substantially. This demonstrates that the correlation between MRSA infection rates and linezolid prescribing is not solely due to increased use of linezolid after linezolid was FDA approved. However, further research is needed to determine why the correlation between clindamycin prescribing and national MRSA outpatient infection rates was influenced by time. Potential reasons for changes in clindamycin prescriptions over time include replacement of clindamycin by other antimicrobials, use of clindamycin for infections other than MRSA, concern in regards to the association between antimicrobial use and *Clostridium difficile* infections, or improved antimicrobial stewardship in the outpatient setting.

Our findings are consistent with those of other studies and the recent CDC Active Bacterial Core findings that community-onset MRSA rates remained steady over time, even though rates of healthcare-onset MRSA have been declining in recent years [7-10,17]. In fact, that CDC study found that for the first time, community-onset MRSA infection rates have surpassed healthcare-onset MRSA rates [17]. Thus, it is more important than ever to provide outpatient clinicians with the resources they need to prescribe appropriate antibiotics in the outpatient setting.

This study is limited by its ecologic study design. Some patients may have received MRSA-directed antimicrobial therapy without having a culture sent to the laboratory, thus they would be represented in the IMS Health Xponent^TM^ database but not in TSN database. However, the IDSA guidelines recommend that cultures be collected and tested from abscesses and other purulent skin and soft tissue infections in patients treated with antimicrobial therapy [2,3]. Thus, patients should be included in both databases. Additionally, MRSA infection was measured by positive culture results rather than the more stringent Centers for Disease Control and Prevention (CDC) National Healthcare Safety Network (NHSN) criteria. However, a study by Harris et al., found that 82% of MRSA positive clinical cultures were MRSA infections as defined by CDC NHSN criteria [18]. Therefore, the majority of clinical cultures in our dataset should be true MRSA infections, not laboratory contaminants. Also, the laboratories included in TSN were chosen to be nationally representative of hospitalized patient populations but may not be representative of all patients receiving outpatient care for wound infections. These laboratories may be located in more urban settings in which MRSA is more likely to be suspected compared with rural settings.

Although national rates of MRSA infections were statistically significantly associated with state-level prescribing of clindamycin or linezolid, the R^2^ values were not exceptionally high (22% and 17%), meaning that the majority (78% to 83%) of variation in antimicrobial prescribing was due to factors other than national rates of MRSA infection. Other factors that would contribute to variation in antimicrobial prescribing include patient signs and symptoms, patient risk factors and severity of infection [2,3]. All of these factors should contribute to therapy decisions. Clinician access to geographic variations in rates of MRSA infections would be just one more tool to assist in the determination of which empiric therapy to prescribe.

We performed this study in order to generate a hypothesis as to whether clinicians rely on local or national MRSA data when prescribing antimicrobials. Future studies should survey clinicians in order to determine what factors are considered when prescribing empirical antimicrobials for suspected wound infections. In summary, clinicians may be relying on national, not local data, when prescribing antimicrobials for suspected wound infections. Local efforts to disseminate local MRSA rates to clinicians may improve empiric prescribing of antimicrobials and should be considered for study in future intervention trials.

## Competing interests

The authors declare that they have no competing interests.

## Authors’ contributions

MLS participated in the study design, participated in the statistical analysis, and wrote the manuscript; ENP, MRE, NB, and RL participated in the study design and helped draft the manuscript; XC and MDS participated in the statistical analysis and helped draft the manuscript. All authors read and approved the final manuscript.
